# Snake Venom Extracellular vesicles (SVEVs) reveal wide molecular and functional proteome diversity

**DOI:** 10.1038/s41598-018-30578-4

**Published:** 2018-08-13

**Authors:** Victor Corassolla Carregari, Livia Rosa-Fernandes, Paulo Baldasso, Sergio Paulo Bydlowski, Sergio Marangoni, Martin R. Larsen, Giuseppe Palmisano

**Affiliations:** 1 Department of Biochemistry, Institute of Biology (IB), Faculty of Medical Sciences, State University of Campinas (UNICAMP), Campinas, SP, Brazil; 2 GlycoProteomics Laboratory, Department of Parasitology, ICB, University of São Paulo, São Paulo, Brazil; 3 Department of Biochemistry and Molecular Biology, University of Southern Denmark, Odense, Denmark; 4 Laboratory of Genetics and Molecular Hematology (LIM31), University of São Paulo Medical School (FMUSP), São Paulo, Brazil

## Abstract

Proteins constitute almost 95% of snake venom’s dry weight and are produced and released by venom glands in a solubilized form during a snake bite. These proteins are responsible for inducing several pharmacological effects aiming to immobilize and initiate the pre-digestion of the prey. This study shows that proteins can be secreted and confined in snake venom extracellular vesicles (SVEVs) presenting a size distribution between 50 nm and 500 nm. SVEVs isolated from lyophilized venoms collected from four different species of snakes (*Agkistrodon contortrix contortrix*, *Crotalus atrox*, *Crotalus viridis* and *Crotalus cerberus oreganus*) were analyzed by mass spectrometry-based proteomic, which allowed the identification of proteins belonging to eight main functional protein classes such as SVMPs, serine proteinases, PLA_2_, LAAO, 5′nucleotidase, C-type lectin, CRISP and Disintegrin. Biochemical assays indicated that SVEVs are functionally active, showing high metalloproteinase and fibrinogenolytic activity besides being cytotoxic against HUVEC cells. Overall, this study comprehensively depicts the protein composition of SVEVs for the first time. In addition, the molecular function of some of the described proteins suggests a central role for SVEVs in the cytotoxicity of the snake venom and sheds new light in the envenomation process.

## Introduction

Snakes are an important group of vertebrates with more than 3000 species^1^, currently distributed among 24 families^2^. Snakes are of great human interest due to the existence of many dangerously venomous species which are responsible for tens of thousands of human deaths per year^3^. All the venomous species have a venom gland that synthesizes, stores and secretes a complex mixture of biological molecules including proteins. Toxins with hemorrhagic and myonecrotic activity are generally found in the venoms of the *Viperidae* family due to synergic action of proteolytic enzymes, such as metalloproteinases and serine proteinases^4^. Such venom components are formed in the venom gland as pre-pro-proteins containing a signal peptide that, once cleaved, produces a mature protein released in its soluble form^5^. After analysis of deduced amino acid sequences from cloned cDNAs of dipeptidyl peptidase IV (DPP IV) and aminopeptidase A (APA), it was noticed that DPP IV and APA are type II membrane proteins that have one transmembrane domain near the N-terminus. N-terminal amino acid sequence analysis of the purified DPP IV revealed that the N-terminus is not processed. This molecular feature raised questions regarding the mechanism of protein secretion into venom^5–7^, which opened up the possibility of alternative routes for snake venom proteins secretion.

This conventional form for venom protein secretion was challenged due to observation of venom proteins with N-terminal transmembrane domains^5–7^, which opened up the possibility of alternative routes for snake venom proteins secretion.

Produced by a variety of cell types, extracellular vesicles (EVs) are able to transfer lipids, nucleic acids and proteins to recipient cells. Depending on the size and the origin, EVs can be classified as microvesicles and exosomes^8,9^. Microvesicles are formed by outward budding and fission of the plasma membrane^10–12^, whereas exosomes are formed intracellularly by inward budding of endocytic compartments membrane, leading to vesicle-containing endosomes, called multivesicular bodies (MVBs)^10^. MVBs eventually fuse with the plasma membrane, thus releasing their internal vesicles (i.e., exosomes) into the extracellular space^10,11,13^. EVs are involved in many biological processes such as cell to cell communication, apoptosis rescue and immunological responses^8,14–16^. Moreover, they have been described to be highly involved in several pathological conditions such as cancer^17^, neurological^18,19^, cardiovascular^20^ and infectious diseases^21^. EVs have been isolated from different organisms from bacteria^22^ to humans^23^ and from different biofluids such as blood, saliva, semen and breast milk^24–29^ using various analytical methods^30^. The presence of vesicles in the luminal face of secretory cells of snake venom glands has been observed since 1973^31^ and three seminal studies have shown the presence of EVs, called microvesicles or exosome-like particles, in snake venom^5,32,33^. Despite a morphological characterization of EVs in snake venom freshly collected, comprehensive studies on the biological content of these vesicles have never been conducted.

Here we use mass spectrometry-based proteomics strategies to assess the protein content in these vesicles and we show for the first time: 1) the isolation of SVEVs from lyophilized venom, 2) the characterization of their morphological features, 3) the composition of their protein cargo and 4) their biochemical and cellular activity. The SVEVs analyzed in this study were isolated from lyophilized venoms from *Agkistrodon contortrix contortrix, Crotalus cerberus oreganus, Crotalus atrox and Crotalus viridis* species. Overall, this study shed new lights on SVEVs protein cargo and their function and proposes them as additional important players in the envenomation process.

## Results and Discussion

### Isolation and characterization of secreted vesicles from snake venoms

**Figure 1:**
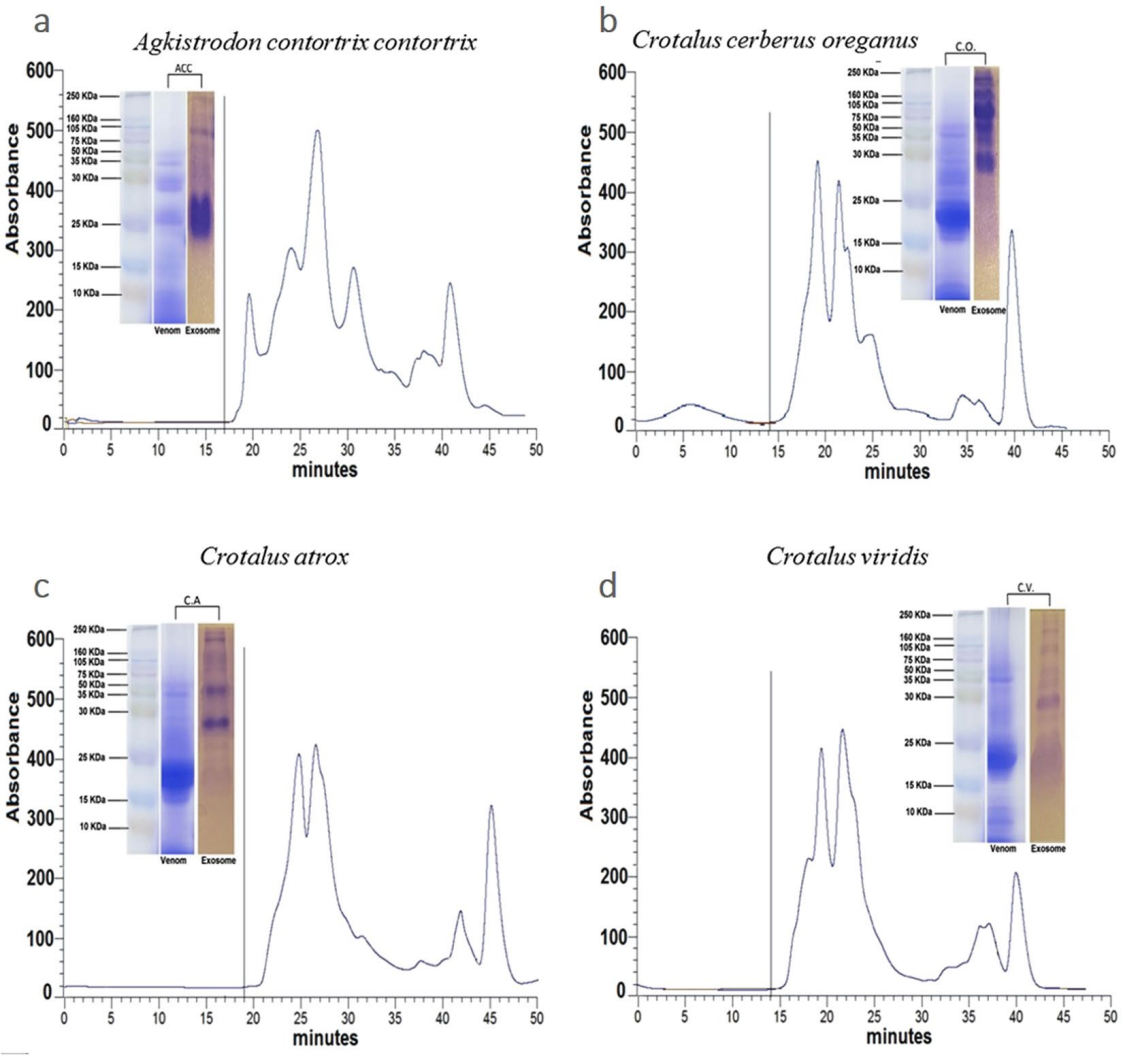
Size Exclusion Chromatographic profile of the whole venom. Chromatographic profiles of the whole venom were obtained for each snake species using size molecular exclusion on Sephadex G75. Only the unretained fractions (indicated by grey line) were collected from each venom Agkistrodon contortrix contortrix (**a**), Crotalus cerberus oreganus (**b**), Cotalus atrox (**c**) and Crotalus viridis (**d**). The insert of each panel shows the SDS-PAGE separation of the proteins collected in the unretained fractions (lane 1) compared to the whole venom (lane 2). Molecular markers are reported on the side of the gel.

The isolation and characterization of morphological and molecular features of SVEVs were performed using a comprehensive experimental strategy as reported in Supplementary Fig. 1. Size exclusion chromatography (SEC) is a largely applied approach to isolate and purify snake venom proteins. However, the composition of the unretained fraction from the SEC column has been ignored and poorly characterized due to the low absorbance in the classical UV range. Despite the low 280 nm absorbance in the SEC column flow-through, we collected this unretained fraction and compared it with the retained fraction from crude lyophilized venom from four different snake species: *Agkistrodon contortrix contortrix (A.C.C), Crotalus cerberus oreganus (C.O),Crotalus atrox (C.A) and Crotalus viridis C.V)* (Fig. 1a–d). In order to visualize the protein content of the unretained fraction, SDS-PAGE was performed and compared with the total venom for each species (Fig. 1a–d**)**. Interestingly, the unretained fractions showed bands with proteins in a wide MW range from 10 kDa to 200 kDa. The SDS-PAGE protein pattern was different between the unretained fraction and the whole venom, suggesting a concentration/enrichment of particular proteins in the non-retained fraction. These first evidences suggested the presence of larger proteins complexes or extracellular vesicles (EVs) in the lyophilized snake venom. The presence of protein complexes was excluded since the proteins were separated under reducing and denaturing conditions. On the other hand, secreted fluids are known to be rich in EVs, which can be released from different cells of the organism. The EVs have a double layered membrane that protects their protein cargo, thereby allowing them to be carried over distances^13^ and exchanged between EVs-producing and target cells^14–16^. Several methods have been used to isolate EVs, where ultracentrifugation (UC) and SEC are among the most applied ones^34^. Two manuscripts have shown EVs isolation from freshly extracted venom from *Crotalus durissus terrificus* and *Gloydius blomhoffii blomhoffii* using differential ultracentrifugation and SEC, respectively^5,33^, suggesting that, in this study, the unretained SEC fraction could also contain proteins encapsulated in EVs.

**Figure 2:**
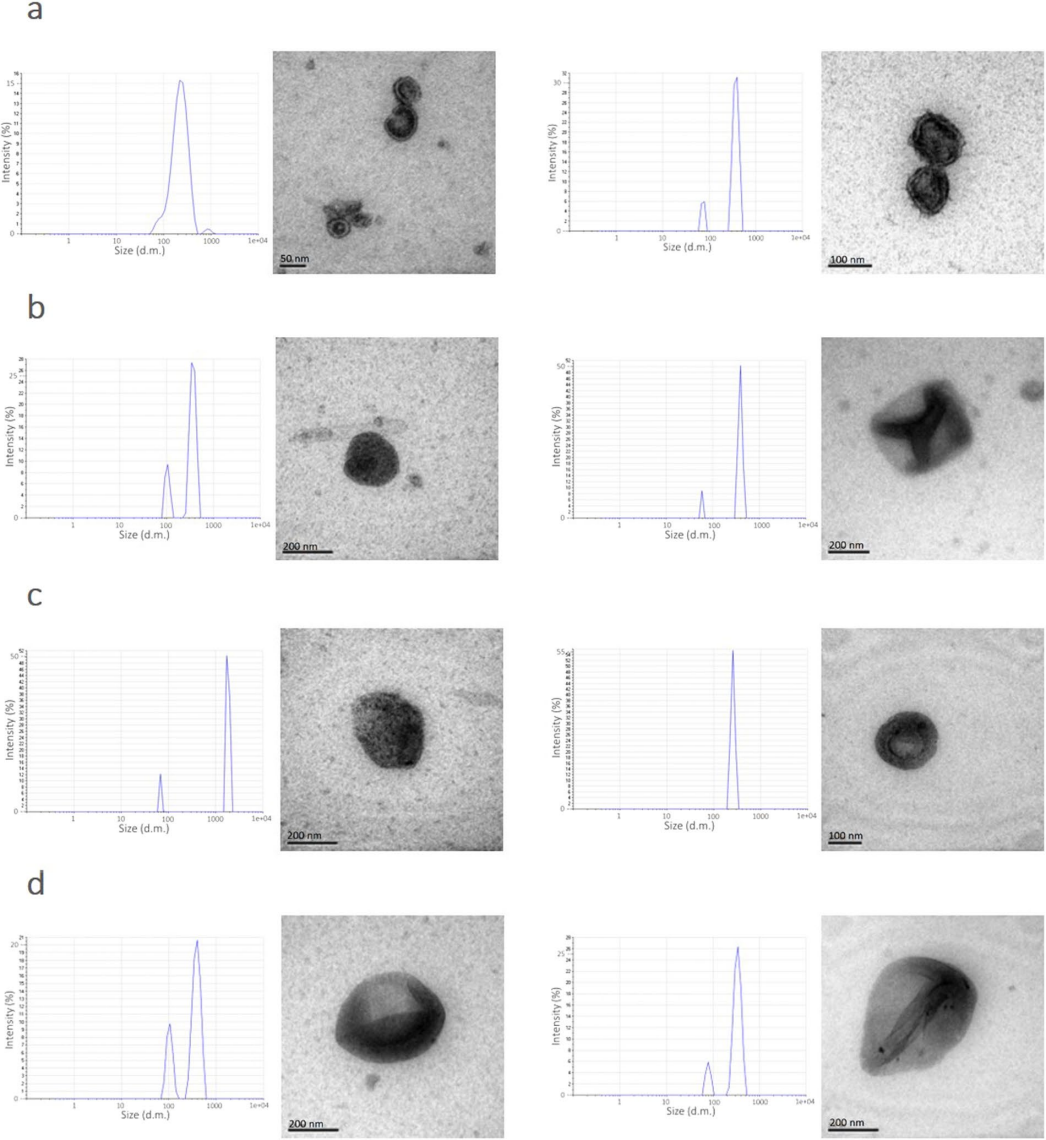
Morphological analyses of the SVEVs. Transmission electron microscopy (TEM) images of vesicles isolates from lyophilized snake venom and size distribution of SVEVs analyzed with dynamic light scattering (DLS). (**a**) Agkistrodon contortrix contortrix (**b**) Crotalus cerberus oreganus (**c**) Cotalus atrox (**d**) Crotalus viridis.

Furthermore, dynamic light scattering (DLS) and transmission Electronic Microscopy (TEM) were performed to validate the presence of EVs. In all venoms analyzed, DLS showed two populations of vesicles with 100 and 500 nm diameter (Fig. 2). It should be noted that Carneiro *et al*.^33^ observed vesicles with 48 nm average size and also vesicles with more than 100 nm and less than 40 nm. Accordingly, Ogawa *et al*.^5^ observed vesicles from 30 to 130 nm in size. Extracellular vesicles^8,9^ have been classified into apoptotic bodies (>1000 nm), microvesicles (100–1000 nm), and exosomes (30–100 nm) depending on their size. The different size of SVEVs could be due to the isolation method, ultracentrifugation or SEC, and especially the use of fresh or lyophilized venom. Indeed, in this study, lyophilized venom was used and this could explain the presence of larger particles (500 nm) that could have been formed during the lyophilization process^35,36^. EVs tends to aggregate, flocculate and degrade during freeze-drying^34,35^ (patent CN104488850A).

Furthermore, TEM was performed on vesicles isolated from the four venoms (Fig. 2). The SVEVs appeared to be structurally intact with a classical exosomal cup-shaped and a lipid bilayer membrane. The size measured using the TEM was in agreement with the DLS data, showing a diameter range from 50 nm to 200 nm (Fig. 2). However, no large particles (500 nm) were observed. In order to isolate SVEVs from the whole lyophilized venom, differential ultracentrifugation was performed as a complementary isolation method. On the isolated material, DLS and TEM analyses were performed, showing similar vesicle diameter and morphological characteristics to the ones obtained by size exclusion (Fig. 2 and Supplementary Fig. 2). Thus, the SVEVs isolated in this study using lyophilized venom share similar morphological characteristics to the ones obtained in the two previous reports.

Snake venom EVs were proposed to contain specific proteinases and prevent their activation inside the gland or direct their action through EVs-mediated delivery systems^33^. The presence of active dipeptidyl peptidase IV, aminopeptidase A and ecto-5′-nucleotidase was also demonstrated^5^. However, the full protein diversity and their biochemical and cellular functions have not yet been revealed. Therefore, a mass spectrometry-based proteomic strategy combined with bioinformatics and functional assays was applied to characterize the SVEVs proteome.

### Proteomic characterization of the SVEVs

**Figure 3:**
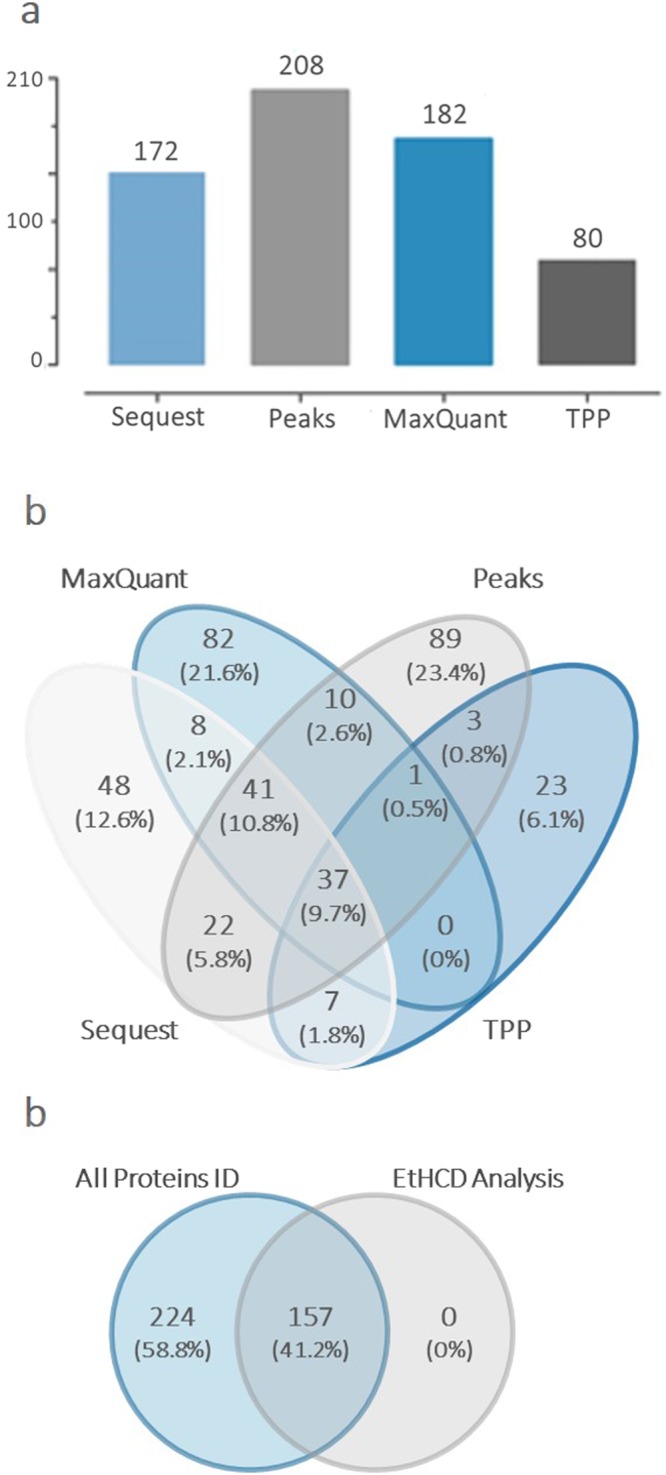
Different search tools provide distinct protein identification. Proteomic analysis of the proteins families for each snake venom species and an overview of all the proteins identifications and search tools analysis (SEQUEST, PEAKS, MaxQuant, TPP and Byonic for the EThcD). (**a**) Number of total SVEVs proteins identified by the each search engine. (**b**) Comparison of total SVEVs proteins identified by each database search engine. (**c**) Comparison of total SVEVs proteins identified by LC-MS/MS with two fragmentation techniques, HCD and EThcD. Byonic software was used to process EThcD data.

**Figure 4:**
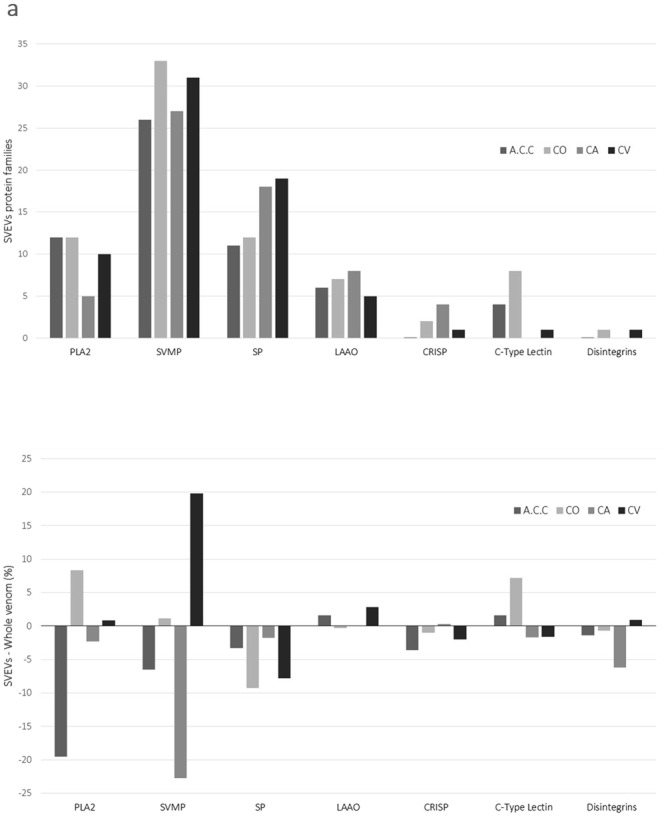
Protein families identified by mass spectrometry-based proteomic analysis of SVEVs. (**a**) Snake venom extracellular vesicular content was analyzed by mass spectrometry-based proteomics revealing similar protein composition between all species. Snake venom metalloproteinase family, serine proteases and phospholipase A2 were among the most represented proteins. Each protein family is reported as percentage (Table 1). (**b**) A direct comparison between SVEVs and whole venom protein families is reported. The difference between the percentages of protein in SVEVs versus whole venom is reported for each family. Bar graph reporting in the x axis the protein families and y axis the difference between percentage of proteins in SVEVs minus percentage of proteins in whole venom for each protein family. The four venoms are reported as (**a**) A.C.C., (**b**) C.V., (**c**) C.O., (**d**) C.A.

Inter and intra-species variation influences the venom composition and the protein concentration^37–40^. Proteomic analyses of venoms, termed “venomics”, have significantly expanded our knowledge and understanding of this biofluid^41,42^, allowing us to better understand the functional aspects of envenomation. In this study, a mass spectrometry-based bottom up proteomic workflow was applied to identify the SVEVs proteome (Supplementary Fig 1). In particular, isolated SVEVs were lysed in urea and proteins digested using trypsin. Subsequently, tryptic peptides were analyzed using high resolution and accuracy mass spectrometry coupled to nanoflow liquid chromatography. Multiple database search engines such as Proteome Discoverer (SEQUEST), Peaks 7.1, MaxQuant (Andromeda), and the Trans Proteomic Pipeline (Comet) were utilized to assign peptide and protein identifications. A total of 706 non-redundant peptide sequences were identified in the four venoms using the four database search engines with 539, 378, 263 and 207 peptides identified using Peaks, Maxquant, TPP and Sequest, respectively (Supplementary Tables 1–20**)**. Based on these peptide sequences, a total of 381 proteins were identified and grouped in eight functional classes including SVMPs, serine proteases, PLA_2_, LAAO, 5′nucleotidase, C-type lectin, CRISP and disintegrin (Figs 3a and 4a).

In particular, for the *A.C.C*., a total of 201 proteins were identified with 20 (10%) in common between the different search engines (Supplementary Fig. 3). For the *C.O*., a total of 85 proteins were identified with 6 (7.1%) proteins in common between the different search engines (Supplementary Fig. 3). A total of 89 proteins were identified in the *C.A*. venom vesicles with 3 (3.4%) proteins in common between the different search engines (Supplementary Fig. 3). For the *C.V*., a total of 169 proteins were identified with 9 (6.3%) in common between the different search engines (Supplementary Fig. 3). All proteins and peptides identified in each SVEV preparation are reported in Supplementary Tables 1–4 and 5–20, respectively. Taken together, these data showed low overlap among SVEVs proteins using different search engines (Fig. 3b). Snakes are able to survive in several habitats. Due to that, various environmental pressures could have promoted micro-evolutionary processes that caused modification of endogenous proteins^43–45^, which developed into different proteoforms from the same protein family. This protein and peptide complexity reflects the wide range of pharmacological effects produced by each venom component. Moreover, many snake species specialize on specific prey leading to selective pressures resulting in the evolution of advantageous venom phenotypes that may vary based on phylogenetic affinities^37,38^, geographic distribution^46,47^, snake age^48^ and diet^49^. Interestingly, a comparison between different search engines resulted in a modest overlap in the identified SVEVs proteins (Fig. 3b and Supplementary Fig. 3). Some proteins were identified with peptides shared between several proteoforms. Since each search engine uses a different strategy to assign the representative protein for each protein group, the overlap between the different search engines was modest. An example is the peptide RKDLLNR assigned to Group III snake venom metalloproteinase. In this case, MaxQuant and Peaks assigned it to different Uniprot IDs, E9KJY6 and E9KJZ0, respectively (Supplementary Tables 5–8). Another example is the peptide HDNAQLLTAIDLDR assigned to Zinc metalloproteinase-disintegrin-like VAP2A. In this case, TPP and MaxQuant assigned it to A4PBQ9 Uniprot ID and Sequest to C9E1R8 Uniprot ID. On the contrary, a comparison between non-redundant peptide sequences identified using different search engines showed high overlap for each venom (Supplementary Fig. 4a–d) and the four venoms combined (Supplementary Fig. 4e). Aiming to confirm the protein identifications obtained, tryptic peptides derived from SVEVs proteins were sequenced using EThcD fragmentation^50^. After database search, 157 proteins were identified with high confidence and compared with all SVEVs proteins identified using HCD fragmentation. A complete overlap between the two datasets was observed providing further confidence to the identifications (Fig. 3c).

### Protein families in snake venom extracellular vesicles

*Viperidae* venoms contain proteins that interfere with hemostasis and with the blood coagulation cascade, ultimately leading to immobilization, killing and pre-digestion of prey. Individual venom may contain well over 100 proteins and endogenous peptides including various proteoforms^51^. These proteins can be classified into 10–15 protein families, such as the enzymatic L-amino acid oxidase (LAAOs)^52^, snake venom metalloproteinases (SVMPs)^53^, phospholipases A2 (PLA2) and serine proteinases^54,55^, as well as the non-enzymatic peptide myotoxins, C-type lectins, cysteine-rich secretory proteins (CRISPs) and disintegrins, among others^37^.

In this study, the most abundant protein families found in all the SVEVs were the metalloproteinases, followed by serine proteinases and phospholipase A_2_. Moreover, L-amino acid oxidase, cysteine-rich secretory protein (CRISP), 5′-nucleosidases, disintegrins and lectins were identified (Fig. 4a). The four venoms showed similar protein families distribution with the SVMPs, Serine Proteinases and Phospholipase A_2_, being present in higher number compared to LAAO, C-type lectins, CRISP and disintegrins (Fig. 4a). These results are in agreement with venomics data in the literature, which showed the presence of these same conserved protein families^56,57^. However, a different number of proteins belonging to the same protein family were identified in SVEVs isolated from the four venoms. Indeed, SVEVs isolated from *A.C.C*. and *C.O*. snake venoms contained a larger percentage of phospholipase A_2_ in comparison with the other venoms studied and with less proteins belonging to the serine proteinases family (Fig. 4a). *A.C.C*. venom has a different protein families profile compared to the *Crotalus* venoms^58^ (Table 1). Indeed, the percentage of PLA_2_ protein family in the *A.C.C*. venom is 30% compared to less than 10% in the *C.O., C.A*. and *C.V*. venoms (Table 1). A direct comparison between the percentages of protein families identified in the SVEVs and in the whole venom showed differences between the four venoms with enrichment of specific protein families in the SVEVs depending on the snake species (Table 1 and Fig. 4b). These data confirmed the various SVEVs protein profiles obtained by SDS-PAGE (Fig. 1). Therefore, SVEVs seem to contain the same protein families as the whole venom although with different representation (Table 1, Figs 1 and 4b), suggesting an important role of these vesicles in the envenomation process.

**Table 1:** Comparison between protein families identified in the SVEVs and in the whole venoms^117^.

**-** ***A.C.C*** *.* **SVEVs**	**%**	**Whole Venom** ^**a**^	**%**
PLA2	12	PLA2	31.5
SVMP	26	SVMP	32.5
SP	11	SP	14.3
LAAO	6	LAAO	4.4
CRISP	0.1	CRISP	3.7
C-type lectin	4	C-type lectin	2.4
Disintegrins	0.1	Disintegrins	1.5
**-** ***C.O*** *.* **SVEVs**	**%**	**Whole Venom** ^**b**^	**%**
PLA2	12	PLA2s	3.63
SVMP	33	SVMPs	31.9
SP	12	SP	21.2
LAAO	7	LAAO	7.3
CRISP	2	CRISP	3.03
C-type lectin	8	C-type lectins	0.78
Disintegrins	1	Disintegrins	1.67
**-** ***C.A*** *.* **SVEVs**	**%**	**Whole Venom** ^**c**^	**%**
PLA2	5	PLA2	7.3
SVMP	27	SVMP	49.7
SP	18	SP	19.8
LAAO	8	LAAO	8
CRISP	4	CRISP	3.7
C-type lectin	N.D.	C-type lectin	1.7
Disintegrins	N.D.	Disintegrins	6.2
**-** ***C.V*** *.* **SVEVs**	**%**	**Whole Venom** ^**d**^	**%**
PLA2	10	PLA2	9.2
SVMP	31	SVMP	11.2
SP	19	SP	26.8
LAAO	5	LAAO	2.2
CRISP	1	CRISP	3
C-type lectin	1	C-type lectin	2.6
Disintegrins	1	Disintegrin	0.1

The data presented for the whole venom have been collected from the literature. All comparisons were made for each corresponding specie as indicated: ^a^^40^; ^b^^116^; ^c^^42^; ^d^^56^. N.D. (not detected). The protein families reported are PLA2: phospholipase A2; SVMP: snake venom metalloproteinase; SP: serine proteases; LAAO: L-amino acid oxidase; CRISP: Cysteine-rich secretory protein; C-type Lectin and Disintegrins.

Interestingly, proteins related to hemostasis or blood coagulation, such as the bradykinin inhibitor peptide and ohanin-like toxin (UniProtKB - P0CJ34 and Q27J48, respectively)^59^ were identified, (Supplementary Tables 1–4). One of the most studied biomolecules isolated from snake venoms are bradykinin and the bradykinin-potentiating peptides. These biomolecules have helped in elucidating cardiovascular physiology^60^. In the literature, several reports on bradykinin-potentiating peptides have been published^56,61,62^. Recently, Sciani *et al*.^63^ identified novel cell penetrating peptides using the low molecular mass fraction of the *Bothrops jararaca* venom. In particular, proline-rich bradykinin potentiating peptide BPP-13a with a C-terminal Ile-Pro-Pro sequence was shown to penetrate human melanoma cell lines without cytotoxicity showing its ability as a cell penetrating peptide^63^. Proteomic analysis of SVEVs identified the bradykinin inhibitor peptide. Little is known about bradykinin inhibitor peptide and its role to antagonize the bradykinin function during vasodilatation^64^. In this study, the bradykinin inhibitor peptide (TPPAGPDVGPR, Uniprot P0CJ34|BKIP_CROAT) was identified in all SVEVs with high confidence suggesting a conserved role for this biomolecule in the biological function of the SVEVs. This peptide has four prolines with two located at the N-terminus suggesting a cell delivery function similar to the bradykinin potentiating peptides.

The identified proteins were classified according to their biological functions, cellular components and molecular functions (Supplementary Fig. 5). The majority of proteins found in the proteomics analyses are present in the extracellular space (57–67%) and cell membrane (10%), indicating an enrichment of secreted proteins. The majority of the proteins are involved in metabolic processes, cell death, response to stimulus and defense response indicating specific roles of the SVEVs proteins. Interestingly, the percentage of SVEVs proteins associated to the different gene ontology categories was similar between the four venoms, indicating a conserved functional SVEVs proteome.

Proteinases represent an important class of proteins identified in SVEVs. Proteinases isolated from the snake venoms are generally classified by their active site into serine proteinases^38^ or metalloproteinases^65^. There are only few evidences for the presence of cysteine proteinases and aspartic proteinases in the venoms^66,67^. Some of them degrade mammalian tissue proteins in the site of bites in a nonspecific manner to immobilize the prey. However, a large proportion of them can cleave plasma proteins of the prey in a relatively specific manner to give potent effects, as either activators or inhibitors, on their hemostasis and thrombosis resulting in blood coagulation, fibrinolysis and platelet aggregation^38,65,68,69^. Serine proteinases are highly represented in the SVEVs proteome suggesting a possible function of these vesicles in the envenomation process.

Another important class of proteinases are snake venoms metalloproteinases (SVMP). These proteins are members of the Reprolysin subfamily of enzymes, which selectively cleave important peptide bonds of basement membrane (BM) components^70,71^, affecting the interactions with endothelial cells^40,72^. The ability of SVMPs to degrade BM components has been known for many years, and it has been hypothesized that hydrolysis of BM proteins is a key event in the onset of microvascular damage and hemorrhage^73,74^. SVMPs are classified into P-I, P-II and P-III classes, according to their domain organization^75^. When comparing the pattern of hydrolysis of BM components *in vivo* and *in vitro* between hemorrhagic and non-hemorrhagic P-I SVMPs from *Bothrops sp*. venoms, a striking difference was found regarding degradation of type IV collagen, as this BM component was hydrolyzed by the hemorrhagic toxin but not by the non-hemorrhagic SVMP^76^. Since type IV collagen plays a key role in the mechanical stability of BM and hence of the capillary vessel structure^77,78^, this observation is likely to have relevant functional implications regarding the mechanism of action of hemorrhagic SVMPs.

SVMPs are also synthesized as zymogens and their prodomain consists on average of 200 amino acids with a conserved sequence, similar to the MMPs and ADAMs prodomains, related to the cysteine-switch mechanism. This process controls the activation state of enzymes by blocking the catalytic site (inactivated state) and proteolytic processing of the prodomain (active state)^79–81^. As showed in previous studies^82–84^ the prodomains of the SVMPs are cleaved after maturation in the Golgi complex or as soon as they reach the lumen of the venom gland. Our proteomic data did not identify any peptide belonging to the prodomain site (Supplementary Fig. 6), suggesting that these proteins could already be released activated in the SVEVs.

### SVEVs show fibrinogenolytic activity

The cargo of the SVEVs include a high concentration of SVMPs and serine proteinases which affect both the hemostatic system and could present catalytic activity on fibrinogen^85^. Most of the venom MMPs, either hemorrhagic or non-hemorrhagic, are fibrin(ogen)olytic enzymes as well as the serine proteinases, cleaving preferentially the α-chain and slowly the β-chain of fibrinogen^86^. Some of the serine proteinases have both fibrogenolytic and fibrinolytic activities. When they have only the fibrinogenolytic activity, they are called ‘thrombin-like’ proteinases, presenting a fibrinogen clotting activity^69,70^. However, during the envenomation process their actions towards fibrinogen as well as other substrates is not exactly identical to those of thrombin. Instead of fibrin(ogen)olytic activity, several venom serine proteinases have the activity for releasing bradykinin from kininogen-like mammalian kallikrein (or kininogenase)^52,87^ and are also called ‘kallikrein-like’ proteinases^87^. In addition, there have been few reports on the venom serine proteinases with a unique activity, such as the activation of factor V^88^, protein C^89^, plasminogen^90^, or platelets^91^.

**Figure 5:**
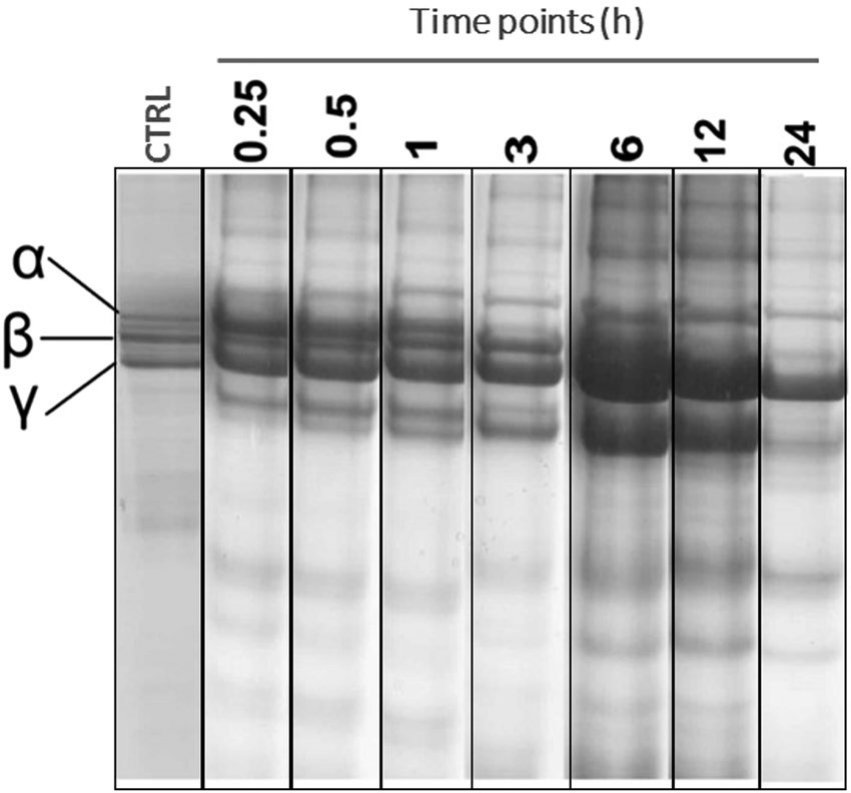
Snake venom EVs present fibrinogenolytic activity. Proteolysis of fibrinogen (2 mg/ml) was assessed by reducing SDS-gel electrophoresis (12%) after incubation with A.C.C. vesicles (15 µg) for different time points (0.25, 0.5, 1.0, 2.0, 6.0, 12 and 24 h). Fibrinogen alone was used as a control.

To evaluate if SVEVs functionally resembles whole venom activity, proteolytic activity of *A.C.C*. SVEVs against fibrinogen was measured. The Aα and Bβ chains of fibrinogen were degraded in a time-dependent manner (Fig. 5). The fibrinogenolytic activity was detected in the Aα chain at 1 h and Bβ chain at 6 h while the γ chain was unchanged (Fig. 5). This assay shows that purified SVEVs have fibrinogenolytic activity. It should be noted that Aα and Bβ chain degradation specificity is not absolute since there is substantial degradation of the alternate chain with increasing time and usually more related with hemorrhagic metalloproteinases^86^. This effect is quite important because it produces an abnormal fibrin clots composed for short polymers that are rapidly dispersed and no longer cross-linked by activated factor XIII, resulting in the disruption of the blood coagulation system of the preys and their immobilization^4^.

### SVEVs presents amidolytic, caseinolytic and esterase activities

In order to further characterize the proteolytic activity of SVEVs isolated from *A.C.C* venom, biochemical assays were performed using azocasein, BApNA and TAME (p-tosyl-L-arginine methyl ester) as substrates^92,93^. Before each proteolytic assay, the sample was submitted to probe tip sonication to release the SVEVs proteins. Every enzymatic assay performed was positive (Table 2), with high activity detected with azocasein substrate (566.53 ± 14.15 U/mg) when compared to others SVMPs^94,95^. The activity towards the BApNA substrate (0.17 ± 0,012 nmol/min) was lower compared to the Rhombeobin serine proteinase^96^. The lower activity found on BApNA substrate could be explained by the cleavage site specificity of the MMPs which require the presence of at least one hydrophobic amino acid, especially Leucine at the position P1′^75,97,98^. Another enzymatic assay was performed using this TAME substrate. The arginine in the TAME substrate have an ester bond with the chromophore while the BApNA have an amide bond between nitroaniline and arginine, therefore the TAME reveals esterolytic activity while the BApNA reveals amidolytic activity^67,99^. We found a moderate activity towards the TAME substrate (Table 2), meaning that the serine proteinases found in the SVEVs have more affinity for the ester bond. This was also found in proteolytic studies of the contortrixobin (Thrombin-like toxin) activity over synthetic substrates^100^ (Table 2).

**Table 2:** Proteolytic activity of the SVEVs with BApNA (N-benzoyl-L-arginine *ρ*-nitroanilide), Azocasein and TAME (Nα-p-Tosyl-L-arginine methyl ester).

Activity	Substrate	SVEVs	Other venom
Amidolytic	BApNA	0.17 ± 0.012 (nmol/min)	1.06 ± 0.106 nmol/min (*Lachesis muta rombeata/ Rhombeobin toxin)*^96^ 0.58117 ± 0.0636 U/mg (*Bothriopsis taeniata*) Porto *et al*., 2007)^118^
Caseinolytic	Azocasein	566.53 ± 14.5 (U/mg) or 0,821 nm (absorbance)	189,89 ± 25,59 U/mg (*Bothrops marajoensis/*BtaHF toxin)^95^ 0,87 nm (absorbance) (*Agkistrodon contortrix contortrix)*^40^
Esterase	TAME	1109.66 ± 0.34 (U/mg)	282.0 ± 22.6 U/mg (*Bothrops jararaca- Bj-PI2 toxin)*^67^ 287 U/mg (*Crotalus durissus cumanesis)*^119^.

Data are expressed as mean ± SD of three independent experiments. The proteolytic activity in SVEVs was compared to the whole venom or a specific toxin as reported in the literature.

### SVEVs and whole venom inhibits viability of HUVEC cells in a dose-dependent manner

**Figure 6:**
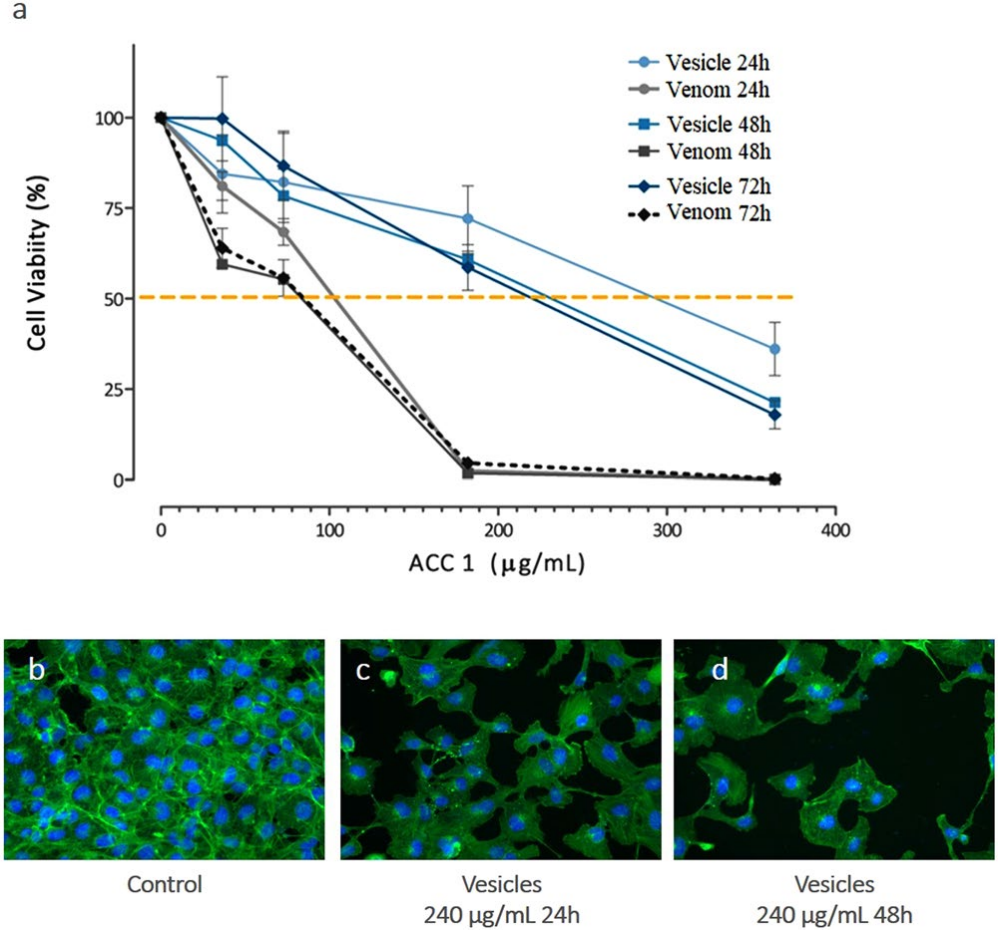
*A.C.C*. whole venom or vesicles obtained by size exclusion chromatography inhibits viability of HUVEC cell line in a dose-dependent manner. (**a**) HUVEC cells were treated with several concentrations of A.C.C. whole lyophilized venom or SVEVs for 24 and 48 h and cell viability was assessed by MTT assay. Data are expressed as mean percentage of cells compared to control with standard errors of at least three independent experiments. (**b**–**d**) HCS analysis of fluorescein-phalloidin staining was used to observe changes in actin filaments organization (green) and nucleus (blue) of Huvec cells without treatment (**b**) and after treatment with A.C.C venom vesicles (240 µg/mL) for 24 h (**c**) and 48 h (**d**).

To assess the cytotoxic activity of *A.C.C*. SVEVs obtained from SEC, as well as whole lyophilized venom, we evaluated the *in vitro* effect on the viability and proliferation of HUVEC endothelial cells by assessing their mitochondrial metabolic activity. Both SVEVs and whole venom inhibited viability of HUVEC cells in a dose dependent manner. Treatment with various concentrations of whole venom showed decreased cell viability in 24 h (IC50 = 86.2 µg/mL), 48 h (IC50 = 58.6 µg/mL) and 72 h (IC50 = 63.2 µg/mL). Treatment of HUVEC cells with increasing concentrations of SVEVs proteins showed reduced cell viability at 24, 48 and 72 h, although the protein concentration required for obtaining 50% inhibition were higher than those observed for the whole venom (IC50 = 279.9, 202.1 and 202.2 µg/mL, respectively) (Fig. 6a–d). The observed difference between vesicle form and whole venom cytotoxicity is not surprising. An early study from Moran and Geren^101^ on *A.C.C*. venom fractions obtained by carboxymethyl cellulose chromatography described that none of the individual fractions presented more cytotoxicity than the whole venom.

To further characterize the effect of SVEVs obtained from SEC on HUVEC cells, changes in actin organization were investigated. After exposure to SVEVs for 24 and 48 h, HUVEC cells show disrupted fiber distribution, loss of intercellular contact and reduction in cell number per site (Fig. 6b–d), highlighting the cytotoxic potential of SVEVs associated with disruption of cell-cell and cell-matrix interactions in consequence of proteolysis of molecules on the cell surface and the extracellular matrix. SVEVs isolated from *A.C.C* venom are rich in proteins from the SVMPs P-II, which have been associated with endothelial cell cytotoxicity accompanied by morphological changes^98,102^. Vascular apoptosis-inducing proteins (VAPs) are toxins capable of triggering apoptosis of vascular endothelial cells associated with cell-detaching activity^103^. It should be noted that the observed cytotoxic effect could also be produced by PLA_2s_ and LAAOs activities. Until now the proportion of encapsulated versus non-encapsulated venom proteins has not been fully elucidated. Ogawa and coworkers noticed that the majority of venom proteins were retained in the size exclusion chromatographic column indicating a low percentage of proteins encapsulated in snake venom vesicles^5^. Andréia Souza-Imberg *et al*. reported that the concentration of proteins in the microvesicles extract was 46.00 µg/mL of fresh venom, indicating a 0.1–0.2% of encapsulated versus total proteins^104^. In our study, we obtained 100 µg of proteins encapsulated in snake venom extracellular vesicles starting from 92 mg of total venom proteins loaded on the SEC column, indicating a 0.1% fraction. These results agree between them and indicate a low percentage of proteins encapsulated in the snake venom extracellular vesicles. The biochemical and biological assays performed in our study confirmed that these vesicles are functional and more studies are needed to evaluate their specific activity.

## Conclusions

In this study, SVEVs were isolated and characterized using a variety of methods including quantitative proteomics. The presence and size of these vesicles was confirmed by DLS and TEM analysis, showing the possibility to isolate extracellular vesicles from lyophilized snake venoms. Proteomic analyses allowed us to identify 381 proteins for all the species studied, belonging to defined functional GO categories such as cell death and defense response, suggesting an important role of these vesicles during the envenomation process. It was showed that the isolated extracellular vesicles are functional. Biochemical and functional assays showed the ability of the SVEVs proteins to hydrolyze synthetic substrates for metallo and serine proteinases and fibrinogenolytic activity. Cytotoxicity assays on HUVEC demonstrated the ability of SVEVs in reducing cell viability and changing actin organization.

Based on the molecular characterization and *in vitro* and *in vivo* functional evidences reported in this study, SVEVs could play an important role in the envenomation of the prey, causing hemostatic imbalance and endothelial degradation, reaching specific cells targets and increasing the efficiency of the prey paralysis. The data presented in this study represent a solid foundation to explore further the role of SVEVs in the envenomation process. Moreover, the proteomic analyses performed in this work could help in finding a possible marker for different species of snakes assisting and revealing new targets for the development of anti-venoms.

## Methods

### Size exclusion chromatography

SVEVs were isolated using size exclusion column (Superdex G75, GE Life Sciences) coupled to an ÄKTA chromatography system equipment (GE Healthcare life science). Fifty milligrams of the lyophilized venom was diluted in 1 mL of 1 M ammonium bicarbonate (AMBIC), centrifuged for 5 minutes at 9000 rpm to remove contaminants such as epidermal cells and other cell debris. The supernatant was applied onto the column and eluted with a pump flow of 0.3 ml/min and 3 mL fractions were collected for each tube in an automatic fraction collector. The unretained fractions from the four venoms were lyophilized and analyzed by Dynamic Light Scattering (DLS) and Transmission Electron Microscopy (TEM). Two SVEVs isolation experiments were performed for each venom and further processed separately.

### Characterization of vesicles

The unretained fractions from the four venoms, obtained by size exclusion chromatography, along with whole venom from *Agkistrodon contortrix contortrix*, *Crotalus atrox*, *Crotalus viridis* and *Crotalus cerberus oreganus*, were characterized by DLS and TEM. In order to concentrate the sample and remove salt interference for further analyses, each fraction was centrifuged at 20,000 × g for 2 h and the supernatant was further centrifuged at 100,000 × g for 2 h. All centrifugation steps were performed at 4 °C to avoid vesicle disruption. Both pellets were collected and vesicles were resuspended in 1 mL of PBS for DLS or formaldehyde fixed for TEM analysis as previously described^105^. Fixed pellets were placed on Formvar-coated copper grids, allowed to adsorb for 20 min, and processed for standard uranyl acetate staining. The grid was washed with PBS and allowed to semidry at room temperature before analysis by TEM (Jeol 1200 EXII). Average hydrodynamic diameter of vesicles dispersed in PBS was determined at 25 °C by DLS using a Zetasizer Nano-ZS (Malvern Instruments).

### Protein digestion and sample desalting

Snake Venom extracellular vesicles obtained from unretained SEC fractions were dissolved in 8 M Urea, 50 mM ammonium bicarbonate (Ambic). Disulfide bonds were reduced by 10 mM DTT at 30 °C for 30 min and cysteines were alkylated by 40 mM iodoacetamide (IAA). Proteins were quantified using Qubit fluorometric detection kit (Thermo Fisher Scientific) and they were digested with porcine trypsin (1:25, w/w) overnight at room temperature. Peptides were desalted using reversed-phase microcolumns packed with Oligo R3 (Applied Biosystem) as described previously^106^.

### Analysis of Snake Venom Extracellular Vesicles by mass spectrometry

Samples were analysed by nanoflow liquid chromatography (Easy nLC, Thermo Fischer) coupled to a Q-Exactive HF (Thermo Fischer) mass spectrometers. Tryptic peptides were separated on a HPLC gradient 2–35% solvent B (A: 0.1% formic acid; B: 100% ACN, 0.1% formic acid) in 30 min at a flow of 250 nL/min. Mass spectrometric analysis was performed using a Q-Exactive HF mass spectrometer (Thermo Fisher Scientific) using a data-dependent acquisition mode. The most intense precursors selected from the FT MS1 full scan (resolution 35,000 FWHM @ m/z 200) were quadrupole-isolated and fragmented by HCD and detected in the Orbitrap mass analyser. The MS1 ion count target was set to 1^6^ (AGC target) and precursor ions were acquired in the mass range of 400–1600 m/z in profile mode with a maximum IT 100 ms. MS2 ion count target was set to 1e5 and the max injection time was 50 ms. Top 12 precursor ions were selected with an isolation window of 2 m/z, fragmented at NCE 30 and detected in the Orbitrap at 17500 resolution. The dynamic exclusion duration was set to 5 s with a 10 ppm tolerance around the selected precursor and its isotopes. Two biological replicates of all the venoms analysed. To further validate peptide identifications, the same samples were analysed on an Orbitrap Fusion Tribrid mass spectrometer (Thermo Fisher) and fragmented using EThcD fragmentation. MS1 spectra were recorded at resolution 120000 in the Orbitrap analyser with a scan range from 600–2000 m/z with quadrupole isolation. The automated gain control target was set to 5 × 105, with a max. injection time of 100 ms. The quadrupole was used for precursor isolation with an isolation window of 2 m/z with an intensity more than 30000. The monoisotopic precursor selection (MIPS) filter was activated. MS2 spectra were recorded at 30000 resolution in the Orbitrap analyzer. Precursor ions were fragmented using EThcD with ETD reaction time set to 75 ms and ETD supplemental collision energy 38.

### Database search

Raw data were analyzed using four softwares with their associated search engines; Proteome Discoverer v2.1 (Thermo Scientific), MaxQuant^107^, Trans-Proteomic Pipeline (TPP) using the Petunia web interface^108^ and Peaks Studio 7.0 (Bioinformatics Solutions Inc.)^109^. Proteome Discoverer searches were performed using SequestHT while MaxQuant uses the embedded Andromeda search engine against the snake Uniprot^110^ protein database (41699 sequences, updated to January 2016) with the addition of 247 protein common contaminants. The following parameters were used: precursor mass tolerance 10 ppm, MS/MS mass tolerance 0.05 Da. Trypsin was selected as cleavage enzyme with strict cleavage specificity at C-terminal to K or R required. Up to two missed cleavages per peptide were allowed. Carbamidomethyl cysteine (+57.02 Da) was set as fixed modification. The variables modifications were methionine oxidation (+15.99 Da) and deamidation of asparagine and glutamine (+0.984 Da). Shared peptide sequences were grouped as grouped accessions proteins. Proteome Discoverer False Discovery Rates (FDR) at peptide spectrum matching (PSMs) level was calculated using the Percolator algorithm with q value equal or less than 0.01. Protein FDR was kept at less than 1% in both search engines. Relative protein expression was calculated using the Precursor Ion node in Proteome Discoverer and the LFQ with “match between runs” with a match time window of 0.7 min and an alignment time window of 20 min using MaxQuant^107^. TPP database search was performing after converting the raw data files into mzXML format using msconvert. Then, the mzXML files were searched using COMET^111^. The snake Uniprot protein database was used as described before. For COMET searches, the precursor ions mass error was set to ±10 ppm and 0.05 Da MS/MS accuracy. Fixed and variable modifications were set as described before. Peptides and proteins were filtered using the PeptideProphet and ProteinProphet with less than 1% probability.

The raw data were also searched using PEAKS 7.0 (Bioinformatics Solutions Inc.) against the snake venom Uniprot^110^ protein database and filtered at a 1% false discovery rate at protein level. Fixed and variable modifications were set as previously described by M oxidation, NQ deamidation were searched for with a precursor ion tolerance of 10 ppm and a fragment ion tolerance of 0.05 Da. EThcD spectra were searched using the Byonic software v.2.6.46 (http://www.proteinmetrics.com/Protein Metrics Inc.). Searches were performed with the following fixed modifications: precursor mass tolerance of 10 ppm, product ion mass tolerance of 0.05 Da, carbamidomethylation Cys, and fully trypsin specific cleavage with a maximum of two missed cleavages. Variable modifications were set as before. Proteins were filtered with a FDR less than 1%. The mass spectrometric and database search features for each peptide sequence are reported in Supplementary Tables 5–20^112^.

### Bioinformatic analysis

Proteins and peptides identified were further analysed using Protein Center software (Thermo Fisher). Cellular components, biological functions and molecular functions gene ontologies were assigned for each snake species. All the proteins identified in the proteomic analyses were compared in different groups regarding the data base search tool used for the analysis using Venny 2.1^113^.

### Fibrinogenolytic activity

Proteolytic activity upon fibrinogen was measured as described by Rodrigues *et al*.^71^ with some modifications. Briefly, 900 µl of Fibrinogen solution (2 mg/ml) in 10 mM Tris-HCl (pH 7.8) were mixed with 20 µl of the sample in the same buffer at 37 °C for different time intervals: 15 min, 30 min, 1, 3 6, 12 and 24 h. Reaction was stopped with 900 µl of a solution containing 10% (v/v) glycerol, 10% (v/v) b-mercaptoethanol, 2% (v/v) SDS, and 0.05% (w/v) bromophenol blue and incubated for 24 hours. Fibrinogen hydrolysis was demonstrated by SDS-PAGE using 12% polyacrylamide gels.

### SDS-PAGE

Sodium dodecyl sulfate-polyacrylamide gel electrophoresis (SDS-PAGE) was carried out on purified vesicles and in the total venom according to Laemmli^114^. The molecular mass markers used were Full Range Rainbow RPN 800 (Amersham ECL Rainbow Molecular Weight Markers- GE).

### Amidolytic Activity and Determination of Kinetic Parameters

Amidolytic activity was measured using the synthetic substrate N-benzoyl-L-arginine *ρ*-nitroanilide (BA*ρ*NA) modified for 96-well plates. The standard assay mixture contained 50 *µ*L of buffer (10 mM Tris-HCl, pH 8.0, 10 mM CaCl_2_, and 100 mM NaCl), 200 *µ*L of substrate solution (1 mM), 10 *µ*L of water, and 10 *µ*L of the released proteins from the vesicles after probe tip sonication in a final volume of 270 *µ*L. The reaction was carried out in a VERSAMAX microplate reader (Molecular Devices Corporation, Sunnyvale, CA, USA) for 30 min at 37 °C, reading the absorbance at 410 nm. The results were expressed as the initial velocity of the reaction (v0) calculated based on the amount of *ρ*-nitroaniline released^92^. The experiments were performed in triplicate.

### Caseinolytic activity

Caseinolytic activity was determined colorimetrically over the substrate azocasein, following the method propose by Wang & Huang^102^. A total of 90 µl of the azocasein solution (5 mg/ml) diluted in Tris-HCl pH 8.0, and mixed with 10 µL of the proteins present inside of the vesicles and incubated for 90 minutes at the temperature of 37 °C. The reaction was stopped by trichloroacetic acid 5% (v/v), after the samples were submitted to 4 min centrifugation 4565 × g. A total of 150 µl of the supernatant was mixed in 150 µl NaOH (0, 5 M) and proteolytic activity was monitored by determination of azo-peptides produced by proteinase catalytic activity on azocasein an absorbance of 440 nm using a VersaMax microplate reader (Molecular Devices Corporation, Sunnyvale, CA). Proteolytic activity unit was defined as the enzyme quantity (mg), which hydrolyze 1 μg per min of azocasein at pH 8.0 and 37 °C^115^. The experiments were performed in triplicate.

### Esterase activity

Esterase activity was assayed using Nα-p-tosyl-L-arginine ester (TAME)^116^. The reaction mixture consisted of 1.5 ml of substrate (1 mM TAME in 0.1 M Tris–HCl, pH 7.8), 1.4 ml of 0.1 M Tris–HCl, pH 7.8, and 0.1 ml of sample. The mixture was incubated for 10 min at 25 °C after which the absorbance was measured at 253 nm. One unit of activity was defined as an absorbance increase of 0.001/min. The experiments were performed in triplicate.

### Cell culture and experimental conditions

HUVEC cells were maintained in DMEM (Sigma-Aldrich) with 10% (v/v) FBS, 100 U/ml penicillin-streptomycin, 3.7 g/L NaHCO_3_ at 37 °C with 5% CO_2_. Stock solutions of whole venom and samples obtained from size exclusion chromatography were prepared in DMEM or PBS prior to use.

### Cell viability assays

Cell viability was determined using MTT colorimetric method. HUVEC cells were seeded at 96 well plates at a concentration of 0.5 × 10^4^ cells and kept in culture conditions for at least 16 h before incubation with whole venom and samples obtained from size exclusion chromatography for 24, 48 and 72 h. After the designated time points, 1.2 mM MTT reagent (Sigma-Aldrich) was added, followed by DMSO 50% (v/v) after 4 h at 37 °C with 5% CO_2_. The experiment was performed using six replicates for each concentration and was repeated three times. The amount of formazan was determined by measuring the absorbance at 570 nm refereed to 630 nm wavelength. For IC50 calculations, survival data were evaluated by variable slope curve-fitting with GraphPad Prism Software (GradPad Software, CA).

### Changes in F-actin organization

In order to investigate the effects of exposure to vesicles obtained from size exclusion chromatography on the actin cytoskeleton, Alexa Fluor 488^®^ Phalloidin (Molecular Probes^®^) was used as a fluorescent dye according to manufactures instructions. Briefly, after 24 and 48 h treatment, cells were fixed in 4% paraformaldehyde, followed by permeabilization with 0.1% Triton X-100 and stained with 3 U/mL Alexa Fluor 488^®^ Phalloidin for 30 min as cell nuclei were counterstained with Hoechst 33342 (Molecular Probes^®^). Experiments were evaluated with a fluorescence microscope ImageXpress Micro High Content System (Molecular Devices). Nine sites from each experiment were analyzed in triplicate using MetaXpress software (Molecular Devices).

## Electronic supplementary material

Supplementary information

Supplementary Tables 1-4

Supplementary Tables 5-8

Supplementary Tables 9-12

Supplementary Tables 13-16

Supplementary Tables 17-20

## Data Availability

The mass spectrometry-based proteomics data have been deposited to the ProteomeXchange Consortium Username: reviewer87997@ebi.ac.uk, Password: Vm3yZch7.
