# Author Correction: Snake Venom Extracellular vesicles (SVEVs) reveal wide molecular and functional proteome diversity

**DOI:** 10.1038/s41598-018-33284-3

**Published:** 2018-10-23

**Authors:** Victor Corassolla Carregari, Livia Rosa-Fernandes, Paulo Baldasso, Sergio Paulo Bydlowski, Sergio Marangoni, Martin R. Larsen, Giuseppe Palmisano

**Affiliations:** 1 Department of Biochemistry, Institute of Biology (IB), Faculty of Medical Sciences, State University of Campinas (UNICAMP), Campinas, SP Brazil; 2 GlycoProteomics Laboratory, Department of Parasitology, ICB, University of São Paulo, São Paulo, Brazil; 3 Department of Biochemistry and Molecular Biology, University of Southern Denmark, Odense, Denmark; 4 Laboratory of Genetics and Molecular Hematology (LIM31), University of São Paulo Medical School (FMUSP), São Paulo, Brazil

Correction to: *Scientific Reports* 10.1038/s41598-018-30578-4, published online 13 August 2018

The Acknowledgements section in this Article is incomplete.

“LR is supported by Finep (1355/10) and CNPq (202077/2015-2). GP is supported by FAPESP (2014/06863-3) and CNPq (441878/2014-8). The Villum Center for Bioanalytical Sciences at University of Southern Denmark is acknowledged for access to advanced mass spectrometric instrumentation.”

should read:

“LR is supported by Finep (1355/10) and CNPq (202077/2015-2). GP is supported by FAPESP (2014/06863-3) and CNPq (441878/2014-8). The Villum Center for Bioanalytical Sciences at University of Southern Denmark is acknowledged for access to advanced mass spectrometric instrumentation.

Prof. Gilberto B. Domont and Prof. Fabio C.S. Nogueira from the Federal University of Rio de Janeiro, Brazil are acknowledged for critical discussion during the revision of the paper.”

