# Supplementary material for: Snake Venom Extracellular vesicles (SVEVs) reveal wide molecular and functional proteome diversity
Source: Sci Rep. 2018 Aug 13;8:12067. doi: 10.1038/s41598-018-30578-4 (PMC6089973; doi:10.1038/s41598-018-30578-4)
Supplement: Supplementary file 1 — Supplementary information [file 41598_2018_30578_MOESM1_ESM.pdf]

SUPPLEMENTARY INFORMATION

# TITLE: SNAKE VENOM EXTRACELLULAR VESICLES (SVEVS) REVEAL WIDE MOLECULAR AND FUNCTIONAL PROTEOME DIVERSITY


Authors:

Victor Corassolla Carregari[1,2#], Livia Rosa-Fernandes[2,3#], Paulo Baldasso[1], Sergio Paulo Bydlowski[4], Sergio Marangoni[1], Martin R. Larsen[3] and Giuseppe Palmisano[2,*]

Affiliation:

[1] Department of Biochemistry, Institute of Biology (IB), Faculty of Medical Sciences, State University of Campinas (UNICAMP), Campinas, SP, Brazil

[2] GlycoProteomics Laboratory, Department of Parasitology, ICB, University of Sao Paulo, Brazil

[3] Department of Biochemistry and Molecular Biology, University of Southern Denmark, Odense, Denmark

[4]Laboratory of Genetics and Molecular Hematology (LIM31), University of São Paulo Medical School (FMUSP), São Paulo, Brazil.

# These authors contributed equally to this work

*To whom correspondence should be addressed

Contact Information:

Prof. Giuseppe Palmisano, Ph.D.: GlycoProteomics Laboratory, Department of Parasitology, Institute of Biomedical Sciences, University of Sao Paulo, Avenida Lineu Prestes 1374, CEP 05508-000, Sao Paulo, Brazil. E-mail: palmisano.gp@usp.br

Supplementary Figures:

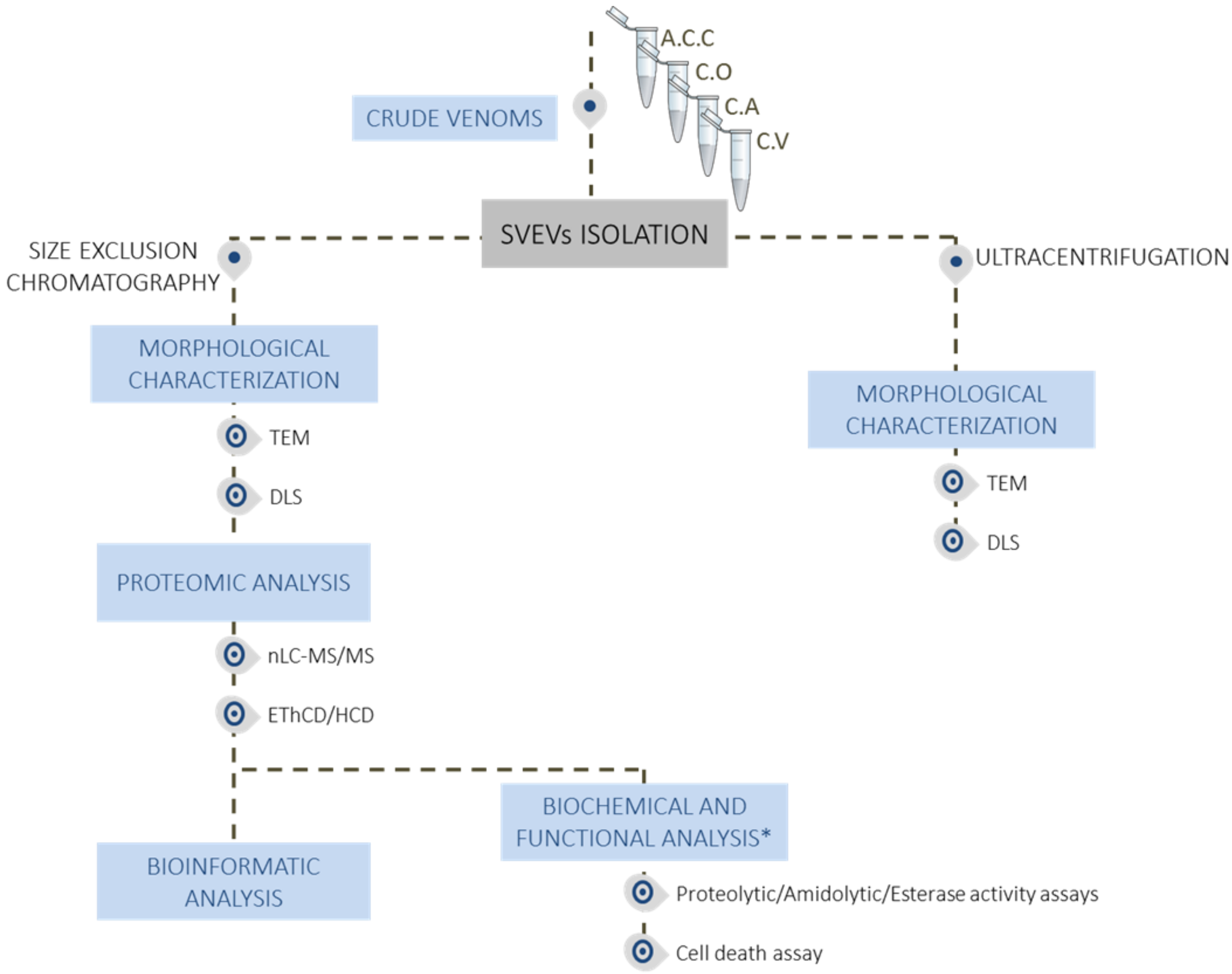


**Supplementary Figure 1: Experimental workflow to elucidate the morphological and molecular features of SVEVs isolated from four snake venoms species** (*Agkistrodon contortrix, Crotalus cerberus oreganus, Cotalus atrox* and *Crotalus viridis)*. Snake venom extracellular vesicles were isolated by size exclusion chromatography and their morphology characterized by transmission electron microscopy and dynamic light scattering. The SVEVs proteome was identified by high resolution mass spectrometry using EThCD and HCD fragmentation. The biochemical activity and cytotoxicity of SVEVs was determined by *in vitro* and *in vivo* assays. SVEVs isolation was also performed using ultracentrifugation as complementary technique before characterization.

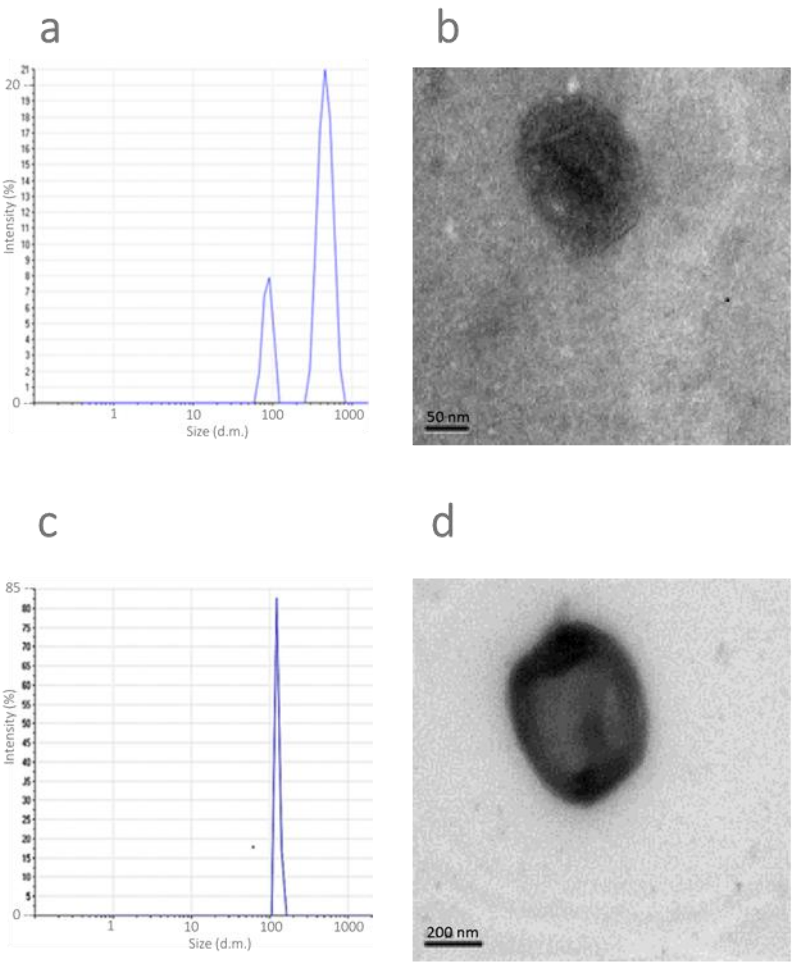


**Supplementary Figure 2: DLS and TEM analysis by the EVs acquired through the ultracentrifugation method of the whole *Agkistrodon contortrix contortrix (A.C.C)* venom. A and B)** SVEVs isolated by ultracentrifugation at 20000xg were analyzed by DLS and TEM. **C and D)** SVEVs were isolated by ultracentrifugation at 100000xg from the supernatant from the first centrifugation and were analyzed by DLS and TEM.

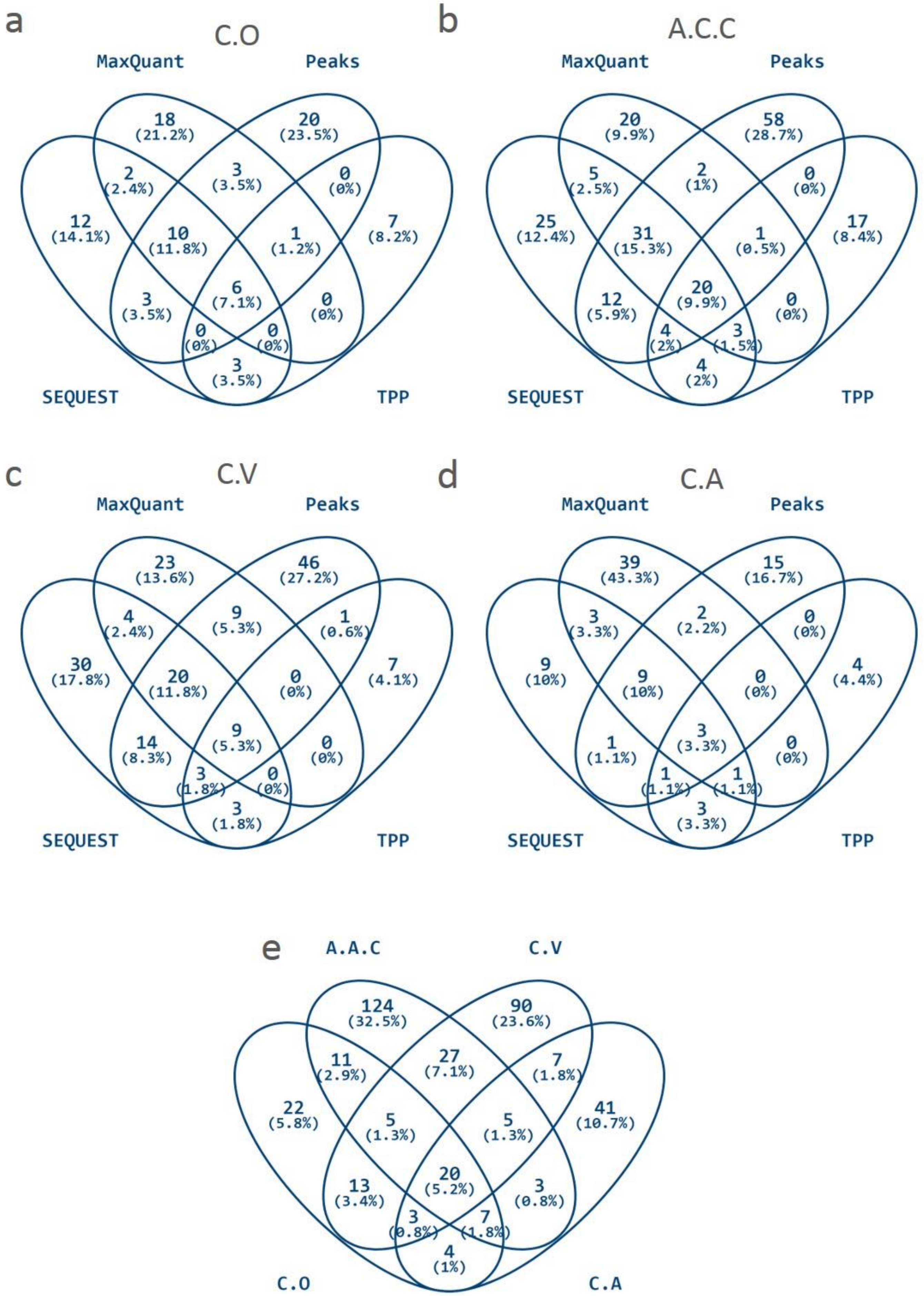
a
C.O
MaxQuant
Peaks
18 (21.2%)
20 (23.5%)
2 (2.4%)
3 (3.5%)
0 (0%)
12 (14.1%)
10 (11.8%)
1 (1.2%)
7 (8.2%)
3 (3.5%)
6 (7.1%)
0 (0%)
0 (0%)
0 (0%)
3 (3.5%)
SEQUEST
TPP
b
A.C.C
MaxQuant
Peaks
20 (9.9%)
58 (28.7%)
5 (2.5%)
2 (1%)
0 (0%)
25 (12.4%)
31 (15.3%)
1 (0.5%)
17 (8.4%)
12 (5.9%)
20 (9.9%)
0 (0%)
4 (2%)
3 (1.5%)
4 (2%)
SEQUEST
TPP
c
C.V
MaxQuant
Peaks
23 (13.6%)
46 (27.2%)
4 (2.4%)
9 (5.3%)
1 (0.6%)
30 (17.8%)
20 (11.8%)
0 (0%)
7 (4.1%)
14 (8.3%)
9 (5.3%)
0 (0%)
3 (1.8%)
0 (0%)
3 (1.8%)
SEQUEST
TPP
d
C.A
MaxQuant
Peaks
39 (43.3%)
15 (16.7%)
3 (3.3%)
2 (2.2%)
0 (0%)
9 (10%)
9 (10%)
0 (0%)
4 (4.4%)
1 (1.1%)
3 (3.3%)
0 (0%)
1 (1.1%)
1 (1.1%)
3 (3.3%)
SEQUEST
TPP
e
A.A.C
C.V
124 (32.5%)
90 (23.6%)
11 (2.9%)
27 (7.1%)
7 (1.8%)
22 (5.8%)
5 (1.3%)
5 (1.3%)
41 (10.7%)
13 (3.4%)
20 (5.2%)
3 (0.8%)
3 (0.8%)
7 (1.8%)
4 (1%)
C.O
C.A

**Supplementary Figure 3: Overlap between all SVEVs proteins identified using different database search engines for each snake venom.** A) *C.O.*, B) *A.C.C.*, C) *C.V.*, D) *C.A.*, E) Combination of the four venoms.

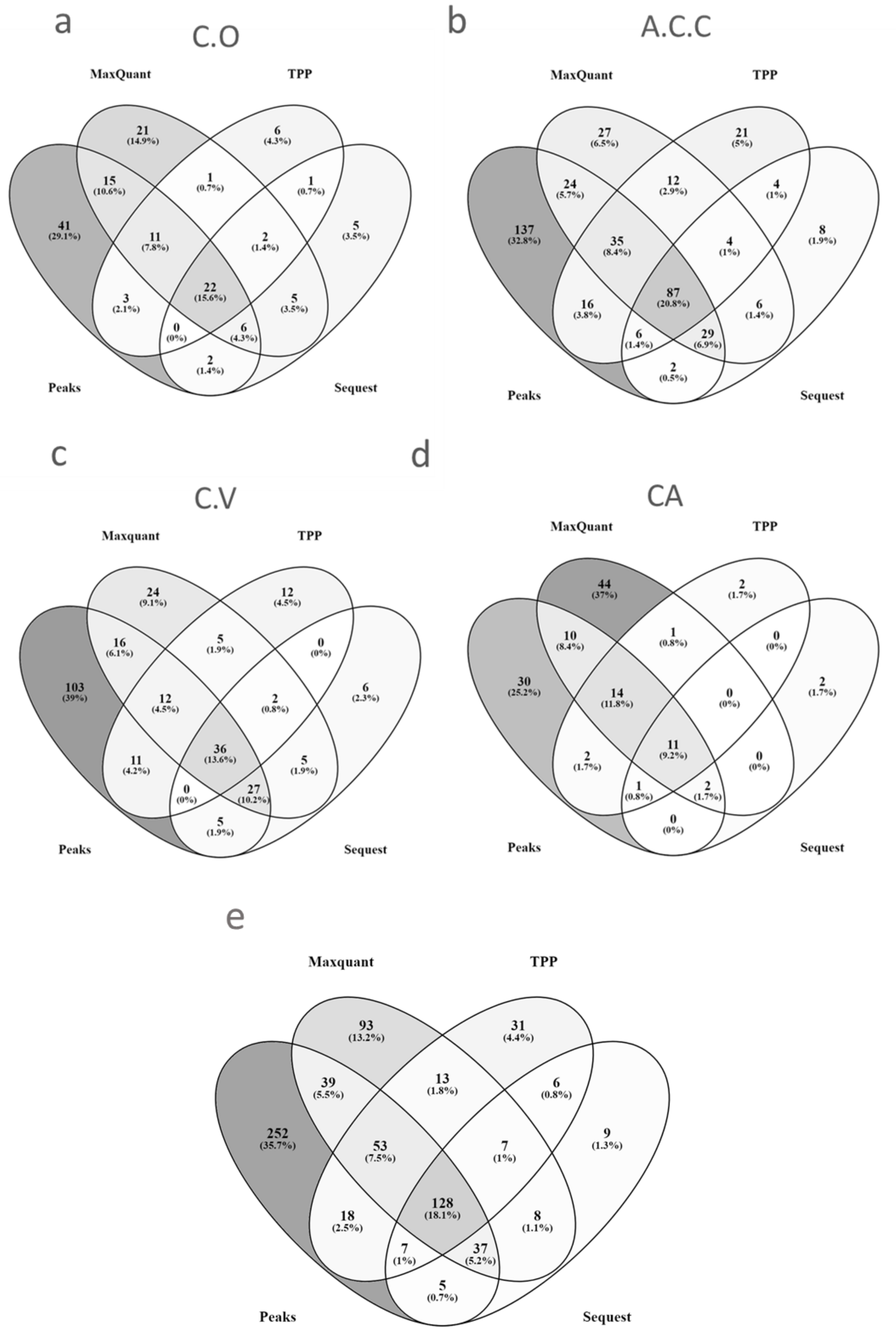

a
C.O
MaxQuant
TPP
Peaks
Sequest
21 (14.9%)
6 (4.3%)
15 (10.6%)
1 (0.7%)
1 (0.7%)
41 (29.1%)
11 (7.8%)
2 (1.4%)
5 (3.5%)
22 (15.6%)
3 (2.1%)
5 (3.5%)
0 (0%)
6 (4.3%)
2 (1.4%)
b
A.C.C
MaxQuant
TPP
Peaks
Sequest
27 (6.5%)
21 (5%)
24 (5.7%)
12 (2.9%)
4 (1%)
137 (32.8%)
35 (8.4%)
4 (1%)
8 (1.9%)
87 (20.8%)
16 (3.8%)
6 (1.4%)
6 (1.4%)
29 (6.9%)
2 (0.5%)
c
C.V
Maxquant
TPP
Peaks
Sequest
24 (9.1%)
12 (4.5%)
16 (6.1%)
5 (1.9%)
0 (0%)
103 (39%)
12 (4.5%)
2 (0.8%)
6 (2.3%)
36 (13.6%)
11 (4.2%)
5 (1.9%)
0 (0%)
27 (10.2%)
5 (1.9%)
d
CA
MaxQuant
TPP
Peaks
Sequest
44 (37%)
2 (1.7%)
10 (8.4%)
1 (0.8%)
0 (0%)
30 (25.2%)
14 (11.8%)
0 (0%)
2 (1.7%)
11 (9.2%)
2 (1.7%)
0 (0%)
1 (0.8%)
2 (1.7%)
0 (0%)
e
Maxquant
TPP
Peaks
Sequest
93 (13.2%)
31 (4.4%)
39 (5.5%)
13 (1.8%)
6 (0.8%)
252 (35.7%)
53 (7.5%)
7 (1%)
9 (1.3%)
128 (18.1%)
18 (2.5%)
8 (1.1%)
7 (1%)
37 (5.2%)
5 (0.7%)

**Supplementary Figure 4: Overlap between tryptic peptide sequences derived from SVEVs proteins identified in each snake venom using specific database search engine.** A) *C.O.*, B) *A.C.C.*, C) *C.V.*, D) *C.A.* and E) Combination of different software for all venoms.

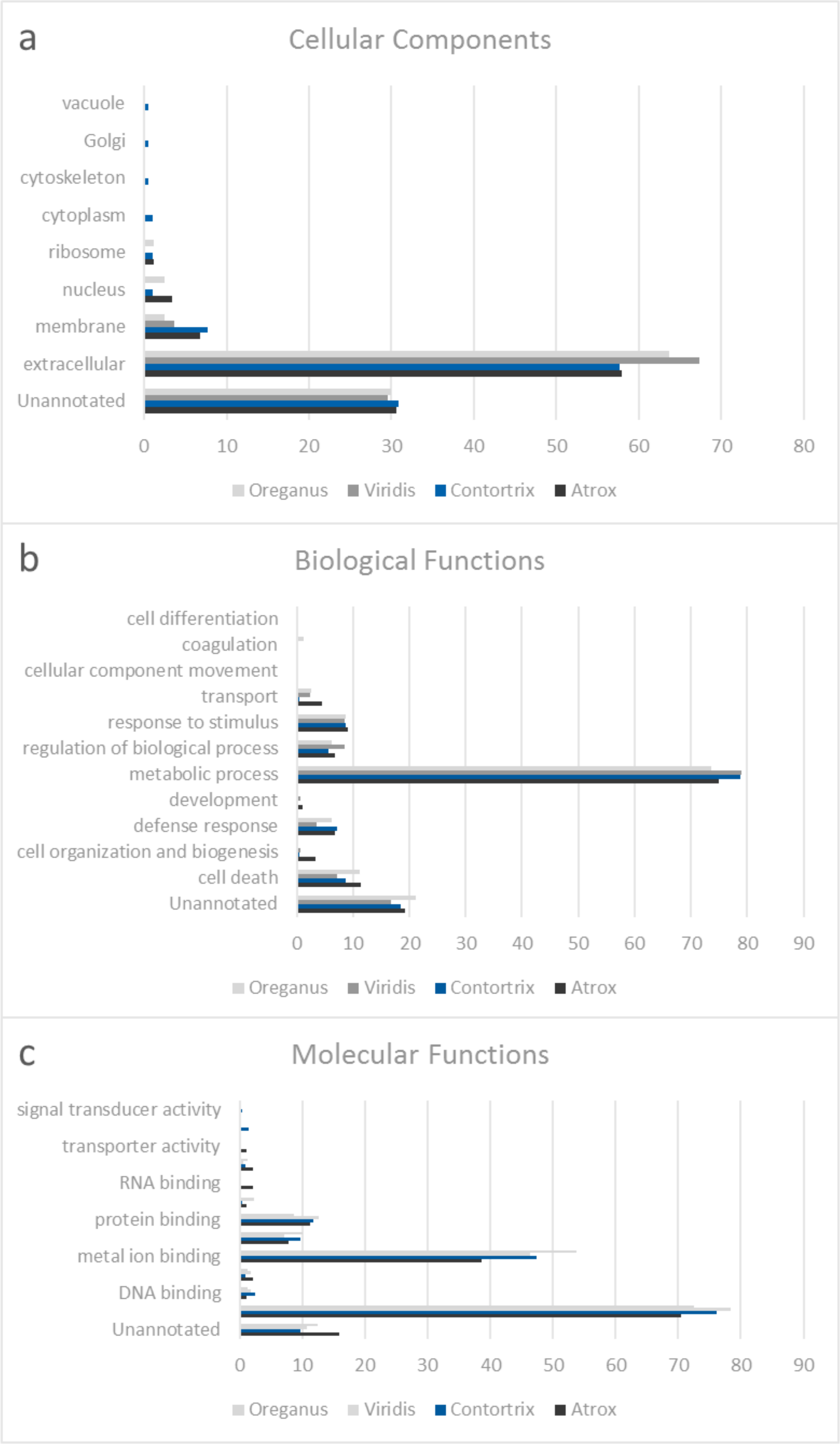


Supplementary Figure 5: Cellular components, Biological Functions and Molecular functions analysis for all the SVEVs proteins.

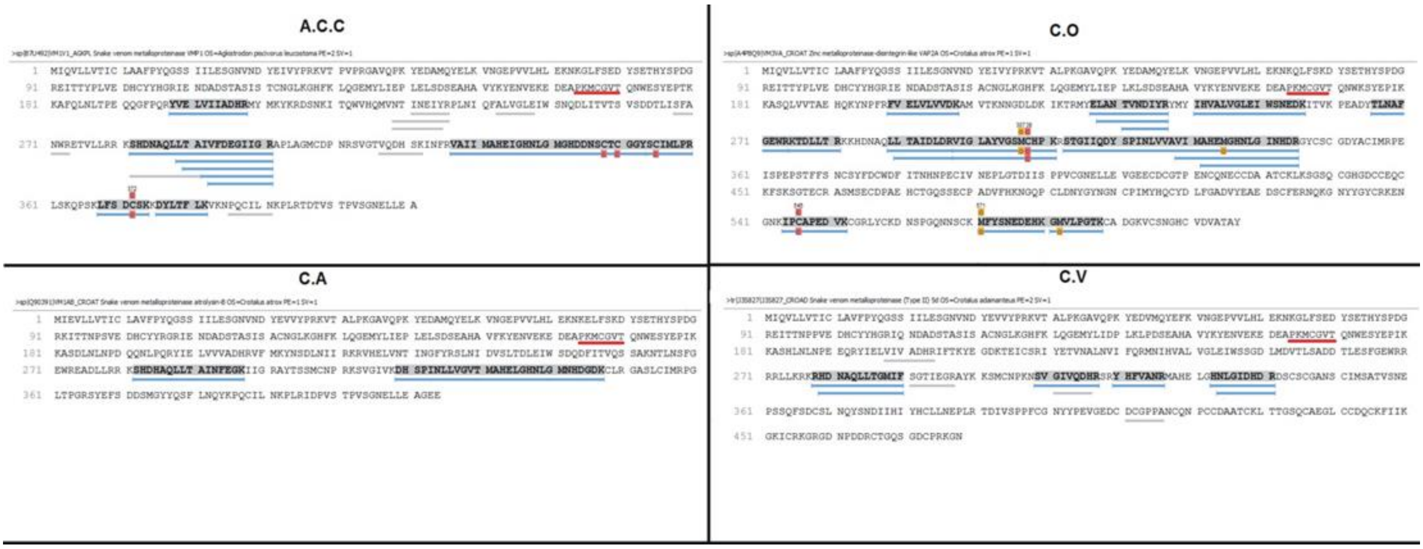

**Supplementary Figure 6-** Snake venoms metalloproteinases identified peptides, showing in a red underlined line a prodomain site.

Supplementary Datasets:

**Supplementary Tables 1-4**. Proteins identified in the SVEVs isolated from four venoms (*C.O., A.C.C., C.V.* and *C.A.*) using the four database search engines: Sequest/Proteome Discoverer (Supplementary Table 1), Andromeda/MaxQuant (Supplementary Table 2), Peaks (Supplementary Table 3) and Comet/TPP (Supplementary Table 4).

**Supplementary Tables 5-8.** Peptides identified in the SVEVs isolated from *C.O.* venom using the four database search engines: Peaks (Supplementary Table 5), Andromeda/MaxQuant (Supplementary Table 6), Comet/TPP (Supplementary Table 7) and Sequest/Proteome Discoverer (Supplementary Table 8). The peptide RKDLLNR highlighted in yellow was selected as an example of protein group assignment as discussed in the main text.

**Supplementary Tables 9-12.** Peptides identified in the SVEVs isolated from *C.V.* venom using the four database search engines: Peaks (Supplementary Table 9), Andromeda/MaxQuant (Supplementary Table 10), Comet/TPP (Supplementary Table 11) and Sequest/Proteome Discoverer (Supplementary Table 12).

**Supplementary Tables 13-16.** Peptides identified in the SVEVs isolated from *C.A.* venom using the four database search engines: Peaks (Supplementary Table 13), Andromeda/MaxQuant (Supplementary Table 14), Comet/TPP (Supplementary Table 15) and Sequest/Proteome Discoverer (Supplementary Table 16).

**Supplementary Tables 17-20.** Peptides identified in the SVEVs isolated from *A.C.C.* venom using the four database search engines: Peaks (Supplementary Table 17), Andromeda/MaxQuant (Supplementary Table 18), Comet/TPP (Supplementary Table 19) and Sequest/Proteome Discoverer (Supplementary Table 20).

Additional information:

This file contains the original SDS-PAGE gels for the four snake venoms and the extracellular vesicles. Moreover, the original SDS-PAGE gel showing the fibrinogenolytic activity is shown below.

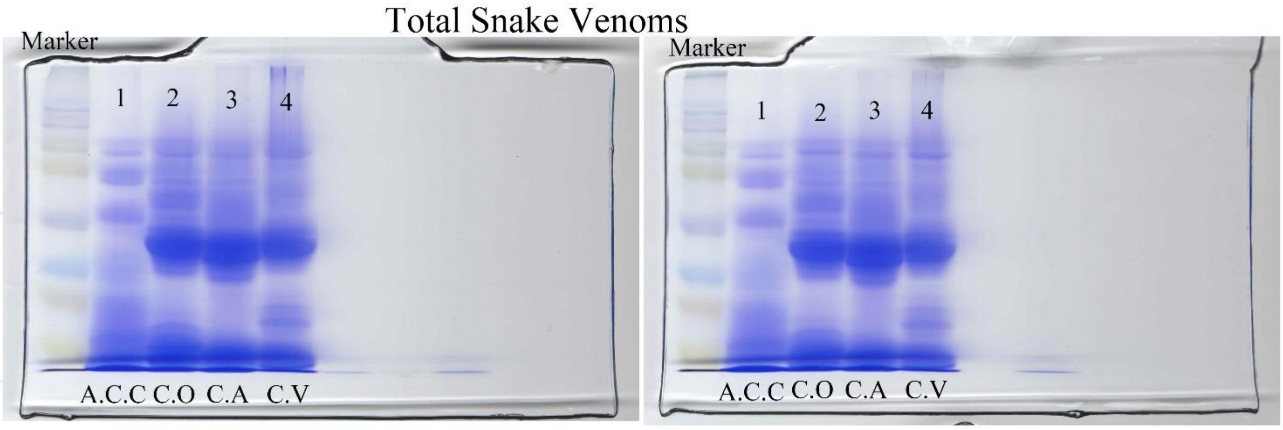


**Additional info 1:** original SDS-PAGE gels of the proteome content of four snake venoms (*ACC, CO, CA and CV*). The experiment was performed in duplicate. Part of these gels were inserted in Figure 1 in the main text.

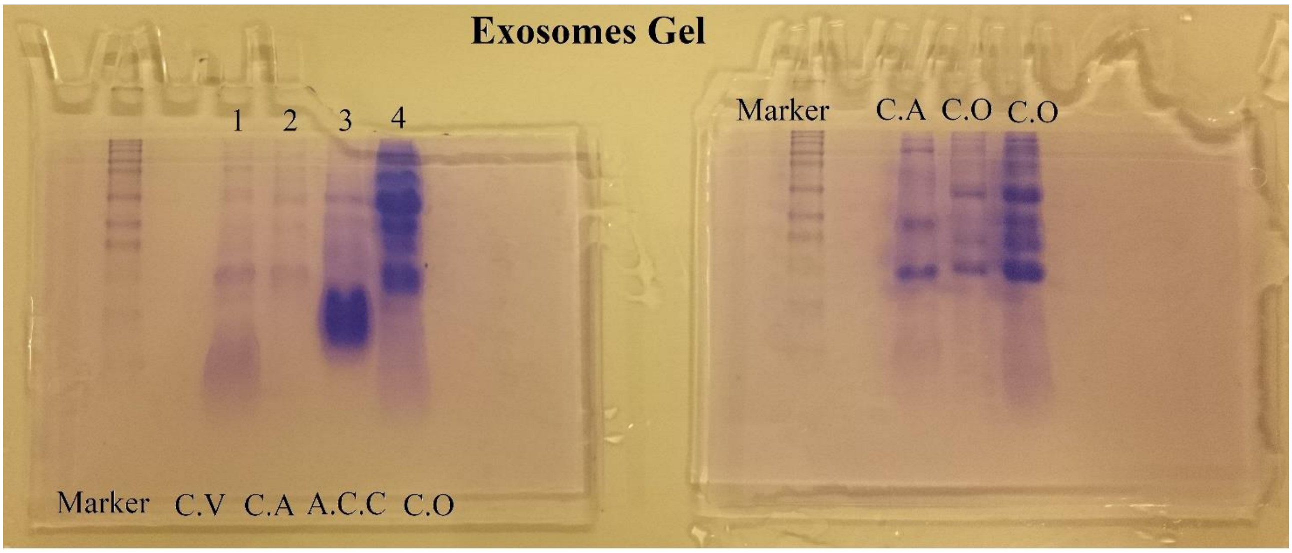

**Additional info 2:** (*left side*) original SDS-PAGE gel of the proteome content of snake extracellular vesicles (SVEVs) isolated from four snake venoms (*ACC, CO, CA and CV*). This experiment was run in duplicate for SVEVs isolated from CA and CO venom (*right side*). Part of these gels were inserted in Figure 1 in the main text.

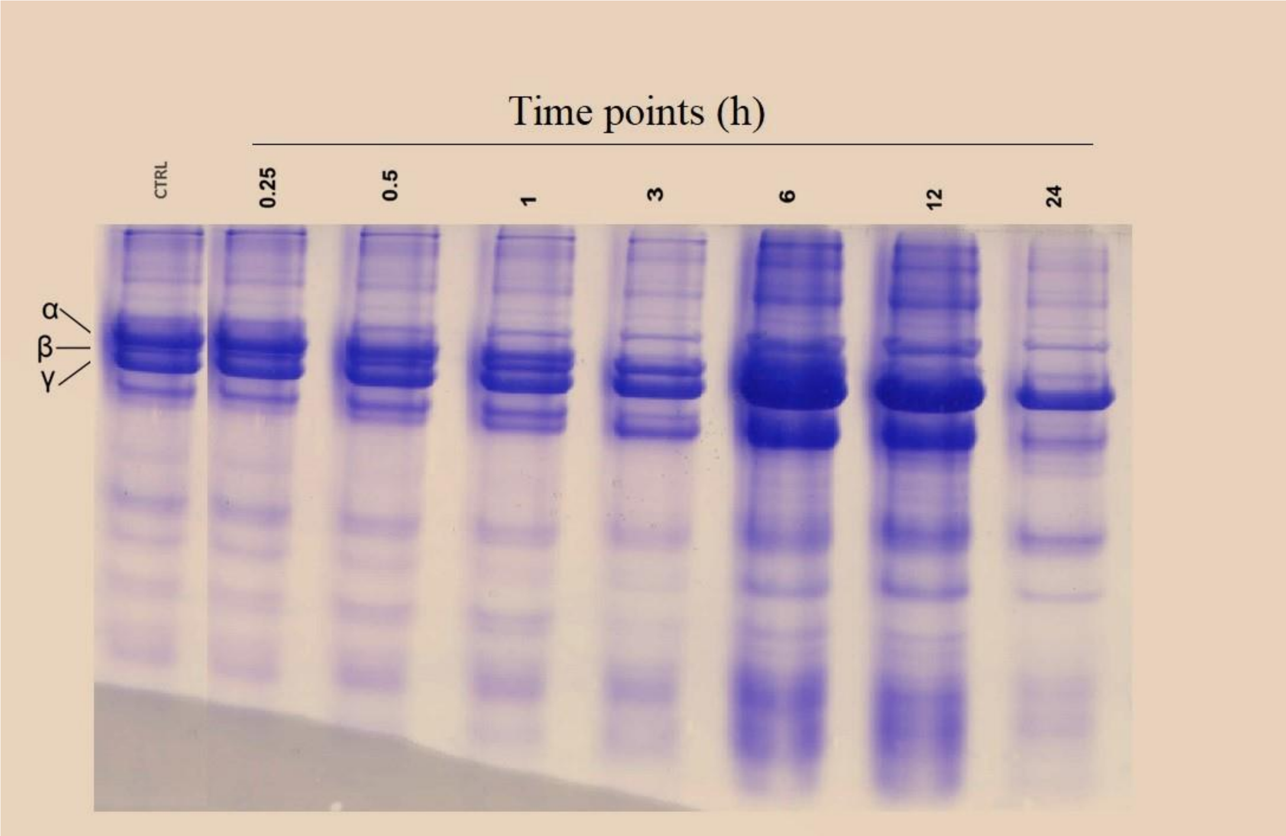


**Additional info 3:** original SDS-PAGE gel showing the proteolytic activity of SVEVs from *ACC* venom upon fibrinogen. This gel is part of Figure 5 in the main text.
